# A multicentre evaluation of the NG-test DetecTool OXA-23 for the rapid detection of OXA-23 carbapenemase directly from blood cultures

**DOI:** 10.1093/jacamr/dlae029

**Published:** 2024-03-07

**Authors:** Alexandra Vasilakopoulou, Thierry Naas, Camille Gonzalez, Jordi Vila, Dóra Szabo, Eleonora Riccobono, Katalin Kamotsay, Sophie Reissier, Dàmaris Berbel, Albert Zoltan Aszalos, Magda Rosenmoller, Milovan Stankov-Puges, Panagiota-Christina Georgiou, Sophia Vourli, Hervé Volland, Spyros Pournaras

**Affiliations:** Laboratory of Clinical Microbiology, Attikon University General Hospital, Medical School, National and Kapodistrian University, Athens, Greece; Team ‘Resist’, INSERM Unit 1184, Faculty of Medicine, Université Paris-Saclay, Bacteriology-Hygiene Unit, Assistance Publique-Hôpitaux de Paris, AP-HP Paris-Saclay, Bicêtre Hospital Le Kremlin-Bicêtre, Paris, France; Team ‘Resist’, INSERM Unit 1184, Faculty of Medicine, Université Paris-Saclay, Bacteriology-Hygiene Unit, Assistance Publique-Hôpitaux de Paris, AP-HP Paris-Saclay, Bicêtre Hospital Le Kremlin-Bicêtre, Paris, France; Department of Clinical Microbiology, Hospital Clinic of Barcelona, Barcelona, Spain; Institute for Global Health (ISGlobal), University of Barcelona, Barcelona, Spain; Department of Clinical Microbiology/CIBER de Enfermedades Infecciosas, University of Barcelona, Madrid, Spain; Institute of Medical Microbiology, Semmelweis University, Budapest, Hungary; Department of Experimental and Clinical Medicine, University of Florence, Florence, Italy; Department of Medical Biotechnologies, University of Siena, Siena, Italy; Central Microbiology Laboratory, Central Hospital of Southern Pest National Institute of Hematology and Infectious Diseases, Budapest, Hungary; Department of Bacteriology, Amiens University Hospital, Amiens, France; Microbiology Department, Hospital de Bellvitge, IDIBELL, UB, CIBERES, Barcelona, Spain; Health Services Management Training Centre, Semmelweis University, Budapest, Hungary; Department of Operations, Information and Technology, IESE Business School, Barcelona, Spain; R&D Department, NG Biotech, Guipry, France; Laboratory of Clinical Microbiology, Attikon University General Hospital, Medical School, National and Kapodistrian University, Athens, Greece; Laboratory of Clinical Microbiology, Attikon University General Hospital, Medical School, National and Kapodistrian University, Athens, Greece; Département Médicaments et Technologies Pour la Santé (DMTS), Université Paris Saclay, CEA, INRAE, SPI, 91191 Gif-sur-Yvette, Paris, France; Laboratory of Clinical Microbiology, Attikon University General Hospital, Medical School, National and Kapodistrian University, Athens, Greece

## Abstract

**Objectives:**

A multicentre study evaluating NG-Test DetecTool OXA-23 for the detection of OXA-23 carbapenemase directly from positive blood cultures (PBCs).

**Methods:**

The NG-Test DetecTool OXA-23 is an immunoassay that integrates a sample preparation device. We evaluated NG-Test DetecTool OXA-23 on 189 spiked and 126 clinical PBCs. The clinical samples’ standard-of-care procedure consisted of bacterial identification from the first day of positivity by MALDI-TOF MS, conventional culture and antimicrobial susceptibility testing. The immunoassay results were verified molecularly. The strains used for the spiked samples consisted of well-characterized *Acinetobacter baumannii* and *Proteus mirabilis* strains.

**Results:**

The NG-Test DetecTool OXA-23 was evaluated on 315 PBCs and revealed sensitivity of 100% (95% CI: 98.21%–100.00%) and specificity of 100% (95% CI: 96.73%–100.00%). It provided 204 true-positive results for OXA-23 in 196 bottles with carbapenem-resistant *A. baumannii* (CRAB) and 8 bottles with carbapenem-resistant *P. mirabilis* and also provided 111 true-negative results. There were no false-positive and no false-negative results. Among the 315 PBCs studied, 83 clinical blood cultures collected in the ICU of a Greek university hospital, which were tested prospectively, all yielded CRAB, and OXA-23 was correctly detected in all samples from the first day of positivity using the NG-Test DetecTool OXA-23.

**Conclusions:**

The NG-Test DetecTool OXA-23 has exhibited excellent sensitivity and specificity for OXA-23 detection in PBCs and can provide valuable information for appropriate selection of antibiotic therapy and early implementation of infection control measures.

## Introduction

The emergence and spread of carbapenemase-producing Gram-negative bacteria (CPGNB) pose a significant global health concern. In addition to carbapenems, CPGNB are usually resistant to many other antibiotics.^1^ Early diagnosis of CPGNB infections is crucial for appropriate treatment and infection control. OXA-23 is the main carbapenemase identified in the *Acinetobacter* genus.^2^ Moreover there have been reports of OXA-23-producing *Proteus mirabilis* from France, Singapore, Belgium, Germany, Finland and other countries.^3–7^ The *bla*_OXA-23_ gene in *Acinetobacter* is most often found in several transposons associated with IS*Aba1* (e.g. Tn*2006* or Tn*2008*) or IS*Aba4* (Tn*2007*).^8^ In *P. mirabilis*, the *bla*_OXA-23_ gene is mainly carried on the chromosome, except for some isolates from France and Singapore that carried *bla*_OXA-23_ on an AbaR4-like structure on an untypeable plasmid.^9^ Due to the association of *bla*_OXA-23_ with mobile genetic elements, it can spread through horizontal gene transfer among microorganisms, and its early detection is of great importance. New diagnostic tests are being developed for the rapid phenotypic or molecular detection of OXA-23-producing Gram-negative bacteria. We conducted a multicentre study that included eight European hospitals for the assessment of an immunochromatographic assay coupled with a filtration/incubation device (NG-Test DetecTool OXA-23) in order to rapidly detect OXA-23 directly from positive blood cultures.

## Materials and methods

### Participating centres

The study took place in eight European microbiology laboratories: Hospital Clinic of Barcelona (Barcelona, Spain), Attikon University Hospital (Athens, Greece), Assistance Publique Hôpitaux de Paris (AP-HP), Paris-Saclay, Bicêtre Hospital Le Kremlin-Bicêtre, (Paris, France), Semmelweis University (Budapest, Hungary), Bellvitge University Hospital (Barcelona, Spain), Florence University Hospital (Florence, Italy), Saint Laszlo-South Pest Hospital (Budapest, Hungary) and Centre Hospitalier Amiens (Amiens, France).

### Study period and samples

The study was conducted from March 2022 till October 2022. We tested 315 positive blood cultures. Eighty-three positive clinical blood cultures with carbapenem-resistant *Acinetobacter baumannii* (CRAB), from different ICU patients, were analysed prospectively in Attikon University Hospital, Athens, Greece. Another 35 positive clinical blood cultures with *A. baumannii* from 35 patients were tested in AP-HP Paris-Saclay, Bicêtre Hospital, Florence University Hospital, Semmelweis University and Amiens University Hospital. One hundred and eighty-one blood cultures spiked with *A. baumannii* were also tested in the participating labs, 62 of which were spiked with *A. baumannii* susceptible to carbapenems, 103 were spiked with OXA-23-producing *A. baumannii*, eight were spiked with OXA-40 carbapenemase-producing *A. baumannii*, 5 were spiked with OXA-58-producing *A. baumannii* and 3 were spiked with NDM-producing *A. baumannii*. All the spiked isolates belonged to the bacterial collections of the Hospital Clinic of Barcelona, AP-HP Paris-Saclay, Bicêtre Hospital and Attikon University Hospital and had been tested molecularly for the presence of *bla*_OXA-23_, *bla*_OXA-40_, *bla*_OXA-58_ and *bla*_NDM_ as described before.^10,11^

Moreover, 16 blood cultures positive for *P. mirabilis* were tested. Among these samples, eight were clinical samples prospectively tested in Florence University Hospital and Saint Laszlo-South Pest Hospital, Budapest (from eight patients) while eight blood cultures were spiked with OXA-23-producing *P. mirabilis* molecularly characterized from the bacterial collection in AP-HP Paris-Saclay, Bicêtre Hospital.^6,10^ For spiking experiments, blood culture bottles considered negative after 5 days of incubation were inoculated with 100 μL of a bacterial suspension containing 10^5^ cfu/mL. Bottles were incubated and tested by NG-Test DetecTool OXA-23 once they were detected as positive by the automatic incubator.

### Identification and susceptibility testing in blood cultures

Blood cultures were incubated using the automated systems BACTEC FX (Becton Dickinson, Hunt Valley, MD, USA) or BacT/ALERT Virtuo (bioMérieux, France). After positivity, Gram staining and subculture to appropriate solid culture media were performed. Identification was accomplished by MALDI-TOF MS (Microflex LT, Bruker Daltonik, Germany or Vitek MS, bioMérieux, France) on the first day of positivity from the broth’s sediment and was verified by MALDI-TOF MS from a colony the next day. Susceptibility testing was performed by disc diffusion directly from the positive blood culture and the semi-automated systems Vitek 2 Compact (bioMérieux, France) or Phoenix 50 (Becton Dickinson). Results were interpreted according to the EUCAST guidelines.

### Sample processing and OXA-23 carbapenemase detection

The OXA-23 detection was performed using the NG-Test DetecTool OXA-23, which is a filtration/incubation device coupled to a lateral flow immunoassay strip (NG-Biotech, Guipry, France). For the clinical samples, the test was performed after the identification of Gram-negative bacteria by MALDI-TOF MS. OXA-23 detection was performed on the first day of blood culture positivity, the processing time was 3–5 min, and the result was read within 15 min. The process, in brief, was: 0.5 mL of the positive blood culture broth was collected from the bottle and mixed with 250μL of cell lysis buffer. After incubation for 2–5 min, 500μL of the lysed sample was transferred to the filtration membrane, and 300 μL of extraction buffer was added. The extract that potentially contained the resistance enzyme then passed through the membrane and migrated through the test device. Two red lines were interpreted as a positive result and one red line in the control region signified a valid negative result. The sample processing device called the BL-DetecTool, has been used previously with other lateral flow immunoassay tests.^12^ The NG-Test DetecTool OXA-23 is a prototype for research use and hasn’t yet been approved for diagnostic use. The positive blood culture broth that was used for the test from the clinical samples was taken from the remaining broth after performing all standard-of-care blood culture procedures on the first day of positivity.

### Molecular methods

Verification of the OXA-23-positive results was done by molecular detection of *bla*_OXA-23_ by PCR and sequencing of selected amplicons.^10^

### Statistical analysis

The calculation and statistical analysis of sensitivity and specificity (with 95% CI) were determined using MedCalc software.^13^

## Results

Overall, the NG-Test DetecTool OXA-23 tested as true positive for the 196 OXA-23-producing *A. baumannii* and the 8 OXA-23-producing *P. mirabilis* and tested as true negative for the 111 OXA-23-non-producing *A. baumannii* and *P. mirabilis* (Tables 1 and 2). The results were verified as true positives and true negatives after molecular testing for *bla*_OXA-23_. In all samples tested, the immunochromatography’s positivity was revealed within 15 min and in most of them in less than 5 min, with easy-to-read strong bands. All immunochromatography results were valid since the control line was present. The sensitivity of the test was 100% (95% CI: 98.21%–100.00%) and the specificity was 100% (95% CI: 96.73%–100.00%).

**Table 1.** Results of the NG-Test DetecTool OXA-23 in blood cultures positive with *A. baumannii*

Hospital	True positive	True negative	False positive	False negative
Attikon Athens, clinical	83	0	0	0
Attikon Athens, spiked	0	17	0	0
HC Barcelona, clinical	—	—	—	—
HC Barcelona, spiked	5	19	0	0
Firenze, clinical	5	0	0	0
Firenze, spiked	—	—	—	—
Paris Saclay, clinical	0	18	0	0
Paris Saclay, spiked	13	19	0	0
Bellvitge, clinical	—	—	—	—
Bellvitge, spiked	27	23	0	0
Semmelweis Budapest, clinical	4	7	0	0
Semmelweis Budapest, spiked	8	0	0	0
Amiens, clinical	1	0	0	0
Amiens, spiked	50	0	0	0
Total	196	103	0	0

**Table 2.** Results of the NG-Test DetecTool OXA-23 in blood cultures positive with *P. mirabilis*

Hospital	True positive	True negative	False positive	False negative
Firenze, clinical	0	4	0	0
Firenze, spiked	—	—	—	—
Paris Saclay, clinical	—	—	—	—
Paris Saclay, spiked	8	0	0	0
South Pest, clinical	0	4	0	0
South Pest, spiked	—	—	—	—
Total	8	8	0	0

## Discussion

This is the first study assessing the sensitivity and specificity of NG-Test DetecTool OXA-23, with the majority of positive results coming from clinical bacteraemic cases. We have shown that this test can serve as a valuable tool in the rapid detection of OXA-23- producing *A. baumannii* and other Gram-negative bacteria directly from positive blood cultures in less than 20 min. The sample processing device (BL-DetecTool), which is coupled to the immunoassay, simplifies the direct testing from the blood culture since there is no need for centrifugation of the sample and the lysed blood culture broth after the extraction step is filtered through the BL-DetecTool. Because of this simple innovative device included in the test, no equipment is needed and the immunochromatographic assay provides a fast and simple-to-read result.

The NG-Test DetecTool OXA-23 test exhibited sensitivity and specificity of 100% in the detection of OXA-23-producing bacteria.

Since OXA-23 is the most prevalent carbapenemase in *Acinetobacter*,^2^ we suggest using this rapid test on the positive blood culture on the first day of positivity after identification of *Acinetobacter* by MALDI-TOF MS on the sediment. Identification by MALDI-TOF MS from the sediment is easy to perform and provides pathogen identification a day earlier. In this study the identification on the first day of blood culture positivity from the sediment was verified by MALDI-TOF MS from the colony on the second day (the results were the same). This is a low-cost method that can provide important information for the adequate treatment of bacteraemic patients a day earlier than the usual conventional blood culture susceptibility testing. The test can be useful also in early detection of bacteraemia by *Proteus* producing OXA-23. Moreover, the rapid detection of patients carrying OXA-23-producing bacteria is important for the timely implementation of infection prevention and control measures. Our results are similar to those of previous studies that had evaluated the use of immunochromatography in carbapenemase detection (KPC, VIM, NDM, IMP, OXA-48) directly from blood cultures.^12,14^ The NG-Test DetecTool OXA-23 can be used for the rapid detection of OXA-23 directly from the positive blood culture bottle after identification of *A. baumannii* or *P. mirabilis*. It can provide early diagnosis, especially in severely ill bacteraemic patients, such as those hospitalized in ICUs, in hospitals where there is a high prevalence of OXA-23-producing CRAB or in patients who had previous known carriage of OXA-23-producing CRAB in surveillance cultures.

There are studies evaluating the use of other immunochromatographic tests for OXA-23 detection using bacterial colonies, namely the OXA-23 K-SeT and RESIST ACINETO (Coris BioConcept, Belgium). Both tests have exhibited 100% sensitivity and specificity for OXA-23 testing from colonies.^15,16^ Nevertheless, our study differs because it evaluates rapid OXA-23 detection directly from positive blood cultures of bacteraemic patients.

A limitation of this study is that the assay was tested in a few *A. baumannii* carrying carbapenemases other than OXA-23. Another limitation is that a clonality investigation of the isolates was not performed. Nevertheless, it is known that most of the CRAB recovered in recent years from highly endemic regions belong to international clone (IC)2 and produce OXA-23 and this was the case also in Greek hospitals^17,18^ and possibly in our setting. In that respect, we believe that the results of the study would be of clinical value for the numerous international regions that are highly endemic for OXA-23-producing CRAB. Of note, the BL-DetecTool can also be used for testing directly for β-lactamases in other samples such as rectal swabs and urine.^13^ At the moment, the BL-DetecTool and the NG-Test DetecTool OXA-23 are ‘research use only’ assays.

In conclusion, the NG-Test DetecTool OXA-23 assay seems to be a reliable, rapid and inexpensive test that could be incorporated in the routine workflow of clinical microbiology laboratories in areas with high prevalence of OXA-23-producing CRAB infections.
